# Intubation of the Larynx, with History of Cases

**Published:** 1885-11

**Authors:** F. E. Waxham

**Affiliations:** Professor of Diseases of Children, College of Physicians and Surgeons of Chicago; 3449 Indiana Ave.


THE CHICAGO

### MEDICAL JOURNAL AND EXAMINER.

VOL. LI. NOVEMBER, 1885. No. 5.

### ORIGINAL COMMUNICATIONS.

INTUBATION OF THE LARYNX, WITH HISTORY OF CASES. *By*
F. E. WAXHAM, M. D., *Professor of Diseases of Children, College of Physicians and Surgeons of Chicago.*

(Read before the Chicago Medical Society, 5th October, 1885.)

At the meeting of this society, March 20th of this year, it was my privilege to present a paper on the "Treatment of Croup." Reference was made to intubation of the larynx and the operation illustrated upon the cadaver. As considerable interest was manifested in regard to this subject, as this interest is leading to practical results, and as several improvements have been made in the laryngeal tubes, I hope I may be pardoned for again calling your attention to this subject.

Intubation of the larynx, as now performed, is due entirely to the patient and long continued investigation, the boldness and ingenuity of DR. O'DWYER, of New York. In the *New York Medical Journal*, of Aug. 8th, he gives a brief and interesting history of his labor in this direction, of his first attempts and of the various changes made in the instruments.

The latest improvements consist, first, in having the tubes made with the head larger and directed somewhat backwards and with the shoulder in the middle of the tube, not rising abruptly from the body, but consisting simply of a gradual increase in the circumference of the tube of this portion.

The enlargement of the head overcomes the danger of slipping into the trachea. The backward curve, given the head, allows the epiglottis to close more perfectly over it, and deglutition becomes easier. The shoulder or enlargement at the center of the tube allows of easier extraction, than when near the head. These instruments now seem about perfect. They are not yet, however, entirely self-retaining. I have but two suggestions to make in regard to them. First, that the tubes be made, if possible, thinner, and second, that they should be made a trifle larger.

Intubation is performed, as has already been illustrated, by having the child held firmly in a sitting posture on the lap of the nurse, with the hands at the side, while an assistant holds the head firmly and somewhat backward. The gag is introduced between the teeth, well back in the left side of the mouth, which the assistant holds it with one hand. The tube, armed with a silk bridle, well waxed, is now secured to the introducing instrument. The right hand manipulates the instrument, while the index finger of the left hand is introduced into the mouth, guiding the tube safely and quickly over the epiglottis and into the larynx. As the introducing instrument is removed, the tip of the finger presses upon the head of the tube and forces it down into the larynx. We now make sure that the tube is in proper position, which will be indicated by easier breathing, by the tube remaining stationary, and by coughing or attempting to swallow water. The bridle of silk is very apt to produce spasmodic coughing and is usually removed. The gag is now re-introduced, the forefinger of the left hand is again introduced over the epiglottis until it touches the head of the tube, when the silk is quickly withdrawn.

This operation, to be performed quickly and successfully, requires practice upon the cadaver. I am informed, however, of a physician who, after extended practice on the cadaver, failed completely in an effort to introduce it in a case of great urgency. With this practice, however, any one who possesses the courage and skill necessary to perform tracheotomy ought to do this operation without serious difficulty.

The removal of the tube is even more difficult than its introduction, as it is almost impossible to hold the child perfectly quiet.

Dr. O'Dwyer advises the use of ether, but I have not yet found it necessary excepting in one case where the tube slipped into the trachea.

It is my pleasure to report five cases of croup treated by intubation. Of this number, one recovered, and another died six days after intubation from pneumonia, the result of unfavorable surroundings. All, with one exception, were unfavorable cases; unfavorable both in regard to age and type of disease. The first patient was two years and one month old, the second three years, the third sixteen months, the fourth, five years, and the fifth two years and seven months. The third and fifth cases were patients with constitutional diphtheria with invasion of the larynx.

I firmly believe that the results in these five cases were better than could have been accomplished by tracheotomy. Indeed, I believe that in but one case would tracheotomy have been justified. In the fifth case, for example, few would have operated upon a child two years and seven months old suffering from constitutional diphtheria. Not only this, but the surroundings positively precluded tracheotomy. The family, crowded in two small rooms, with the child most of the time in the kitchen, its bed a rocking-chair, the cold air entering every time the door was opened, with the most careless and negligent watching and nursing, no one indeed would have thought of tracheotomy under the circumstances; and yet tubing of the larynx prolonged life six days, until the constitutional symptoms had subsided, until the diphtheritic membrane had disappeared from the tonsils and uvula, until the temperature had become normal, and until the child was apparently out of danger. Intubation proved its superiority over tracheotomy in this case. It accomplished all, and more than could have been expected, and death was too plainly the result of unfavorable surroundings.

Speaking from this limited experience, I would advise any one contemplating this operation, first to practice upon the cadaver until he becomes expert in the use of the instruments; second, to possess two sets of tubes, so that if any accident occurs the patient's life may not be sacrificed; third, to always introduce the largest sized tube that can be used.

My first case was reported to this SOCIETY, in detail, at the meeting of April 20th. The patient died thirty hours after intubation.

CASE II. April 23rd. I was called to attend Annie M., aged 3 years, and found her in the last stages of suffocation from pseudo-membranous laryngitis. At 10 A. M. I tubed the larynx without difficulty and with immediate relief to the urgent symptoms.

12 M., Resp. 28, pulse 120, temp. 101½° F.

2 P. M., " 28, " 120, " 101° F. Sleeping quietly.

5 P. M., resp. 28, pulse 130, temp. 99½° F. Coughing considerably. Loose mucous râles. Treatment consisted of bichloride of mercury, one twenty-fourth grain every hour, and mustard draug ts applied every two or three hours to the chest, and oleate of mercury applied externally about the throat and chest, stimulants,—and so forth

9 P. M., pulse 125, respiration 40, temperature 100° F. Bowels moved four times. Mercury omitted.

April 24th, 1 A. M., pulse 130, respiration 52, temperature 100° F. Restless. Bowels moved three times. Opium given and mercury continued.

9 A. M., pulse 130, respiration 52, and of bad character. Removed considerable mucus from the throat, with the finger, causing some relief.

12 M., pulse 130, respiration 48, temperature 99° F. Bowels moved twice. Mercury again discontinued. The tube about this time slipped into the trachea. This accident was indicated by the hoarse voice and by the fact that fluids no longer provoked coughing. Respiration somewhat but not greatly embarassed by this occurence. Made an attempt to remove the tube but could not reach it.

6 P. M. With the assistance of PROFESSOR STEELE, ether was given the child and the tube was removed at the first attempt. It was found in the trachea, just below the vocal cords. Respiration was even more embarassed after the removal of the tube, but the parents objected to its re-introduction and the child died two hours later, thirty-six hours after intubation.

CASE III. Through the courtesy of PROFESSOR HATFIELD, I was called to see a little patient, sixteen months old, in a dying condition, from diphtheritic laryngitis. With the assistance of PROFESSORS HATFIELD, CASSELBERRY and DR. STARKEY, I tubed the larynx with great difficulty. Several attempts were necessary, and it seemed almost impossible to get into the trachea on account of its small size. This case proves the necessity of a still smaller tube than we yet possess, for the tubing of infants under two years. The relief from the urgent and distressing dyspnœa was prompt and complete. In twenty-four hours, however, there was an accumulation of mucus and softened membrane below the tube, that seemed to threaten the infant's life. The tube was removed and large quantities of mucus removed with the finger. The child now seemed to breath easier, but in an hour urgent dyspnœa returned. Another attempt was made to introduce the tube, but it was not crowded down into the trachea far enough, and was soon rejected. As the child was unconscious, and as it was evident that we were only temporizing, no further attempt was made, and the child died an hour later, about twenty-six hours after intubation.

CASE IV. I was called in the evening of September 15th, to perform tracheotomy upon Dora K., a little girl five years old, of delicate constitution, a patient of DR. BEHREND, from whom I obtained the following history. The child had been sick about one week, but had not manifested alarming symptoms until two days previously, when he was summoned, since which time the symptoms of membranous croup were well marked and treatment seemed to have no effect upon its progress. At this time the symptoms were urgent. The voice whispering, the cough hoarse and nearly suppressed, a loud stridor heard on every inspiration, and a deep depression at the base of the thorax with every inspiration. The dyspnœa was indeed distressing to witness, and it was our firm belief that the child could not live long without surgical interference. As the parents were reluctant to have tracheotomy performed, we decided to tube the larynx,—to which they readily consented,—and to perform tracheotomy later if necessary. The father held the child firmly in his arms with the head thrown somewhat backward, and with the assistance of DR. BEHREND the laryngeal tube was introduced quickly and easily, the operation not requiring over twenty seconds. Severe coughing was excited, and a large quantity of thick, tenacious mucus, tinged with blood, was thrown out through the tube. The breathing at once became easy and natural, although every few minutes a severe spasm of coughing occurred which seemed to be caused by the silk which secured the tube. This was removed, leaving the tube in the larynx, to the great astonishment of the parents, who believed we would never be able to remove it. The child now took bread and milk with relish and without difficulty and was left quietly sleeping.

September 16th, 9 A. M., pulse 110, respiration 24, temperature 102° F. Respiration easy, although the tube would occasionally fill with mucus ; however, when urged, she would cough with considerable force, expelling thick mucus, when the respiration would again become noiseless. 9 P. M., pulse 114, respiration 28, temperature 101° F. September 17th, at 7 A. M., during a severe attack of vomiting and coughing, the tube was expelled after being in position about thirty-six hours. The respiration without the tube was now comparatively easy —28 per minute, pulse 120, temperature normal. The little patient was watched very closely, but the tube was not re-introduced. September 18th, 9 A. M., pulse 104, respiration 24, temperature normal. Voice still hoarse, respiration somewhat embarrassed, but no dyspnœa. Slept well during the night and expectorated considerable mucus and softened membrane. She seemed playful and complained of no soreness of the throat. The little patient passed most of the day comfortably, but, towards evening, the voice became more hoarse and croupy, and respiration more embarassed. About 10 P. M., I was summoned in great haste, and found the child again in a critical condition. Respiration was very difficult; again there was the deep sinking in of the tissues at the base of the thorax with every inspiration, pulse 120, and respiration 28 per minute. With the assistance of DR. BEHREND, the tube was quickly reintroduced, not requiring this time over fifteen seconds. Again violent spasmodic coughing was excited and a large quantity of thick, bloody mucus expelled. Spasmodic coughing continued at intervals of a few minutes until the silk securing the tube was removed, when the respiration again became easy and tranquil and the little one was left sleeping.

Sept. 19th, 9 A. M., pulse 100, resp. 24, temp. normal. Slept quietly all night, is playful, no irritation from the tube, and taking nourishment without difficulty. 8 P. M., pulse 120, resp. 24, temp. normal. At 10, P. M., the tube was rejected during a violent attack of coughing and vomiting. Was immediately summoned and found the respiration stertorous, a loud stridor with every respiration, cough husky, and again the deep sinking in of the tissues. A larger tube than had been previously used was now quickly introduced; again violent spasmodic coughing occurred, and large quantities of thick mucus expelled. The silk bridle was removed from the tube, and the result of this simple operation was as wonderful as it was satisfactory. The respiration became noiseless. The loud stridor disappeared, and all the alarming symptoms subsided as by magic. The little patient was again left sleeping quietly.

Sept. 20, 9:30 A. M., pulse 120, resp. 18, temp. normal. Slept well during the night and was bright and playful.

Sept. 21st, pulse 100, resp. 20, and noiseless. The little patient sitting up in the bed, happy with its playthings.

Sept 22d, pulse 120, resp. 20, temp. normal. Only awake twice during the whole night. Considerable thick, ropy mucus expectorated during the morning. As one week had now elapsed since intubation was first performed, during which time the tube had been in the larynx five days, we now thought best to remove it. With the assistance of DR. BEHREND and DR. H. T. BYFORD, an attempt was made to do so, but violent coughing occurred, during which the tube was rejected. Respiration was now carried on comfortably and the tube was not re-introduced.

Sept. 23d, the little patient entirely comfortable, pulse 120, resp. normal, temp. normal. The voice clear and natural, and convalescence seemed assured.

Sept. 27th, child seemed well, but not yet allowed to leave the bed.

Oct. 1st, child about the house and entirely out of danger.

CASE V. Sept. 24th, I was called to see Ida S., two years and seven months old, a patient of DR. RICHARDSON'S, who has kindly furnished me the notes for the following history: The parents first noticed that the child had taken cold on the evening of the 21st. There was a slight cough and the child was feverish and restless. The following day, she complained of sore throat, but took all her food as usual. On the afternoon of the 24th, DR. RICHARDSON was called and found great vascular excitement and much difficulty of breathing. The voice was whispering and the cough stifled, pulse 140, resp. 40, temp. 103°F. On inspecting the mouth, he found diphtheritic deposit on the uvula and velum. He ordered emetic of sulphate of zinc, and, after emesis, a combination of tr. ferri chlor., hydrag. bichlor. and sodii chlorat, every two hours, and trypsin, applied with a brush; at the same time, the steam atomizer with lime water for fifteen minutes every hour.

Shortly after 8 P. M. I saw the little patient, with DR. RICH-

ARDSON, and proposed intubation as the only means of saving her life.

At 10 P. M. we tubed the larynx without difficulty. Immediately after the operation we witnessed the most happy result. The breathing became tranquil and the child fell into a comfortable sleep.

Sept. 25th, the child seemed quite bright and had slept through most of the night; pulse 95, resp. 25, temp. 100 2-5°F. Nourishment and stimulants had been regularly given.

26th, the child appeared not quite so well. Temp. 101°F., pulse 130, resp. 36. There was less false membrane to be seen on the velum and uvula, but there seemed to be some accumulation of mucus and softened membrane in the tube. We thought best to remove the tube, cleanse it, and then re-introduce it if necessary. It was removed with the extracting instruments at the second attempt and without ether. As the child breathed with comparative comfort, we concluded to leave out the tube for a few hours and to crowd stimulants and nourishment.

The tube had been removed at about 8:30 in the evening. We were both called in great haste at 4 A. M. The respiratory efforts were violent, consciousness was impaired, and the child seemed about to suffocate. The tube was immediately re-introduced, and at once the child was relieved and continued comfortable. The little patient for the next two or three days gave every evidence of rapid convalescence, breathing quietly and naturally and taking an abundance of nourishment.

On the morning of the 29th, we found the child again breathing badly and learned that she coughed and vomited in the night. We thought best to remove the tube and made two or three unsuccessful attempts. The tube could not be reached; neither could it be detected with the finger or the extracting instruments, even when passed down below the vocal cords. We began searching about the bedding and clothing of the child and finally discovered it. This accident illustrates the negligence and carelessness, with which we had to contend. The parents were probably asleep when it occurred. If the child had been carefully nursed, this accident could not have occurred without being noticed. The tube was cleansed and re-introduced, giving immediate and great relief. The diphtheritic membrane had now disappeared from the tonsils and uvula.

Sept. 30th, in the absence of DR. RICHARDSON, I was called to see the little patient at 3 A. M., and found marked symptoms of pneumonia. The child was hot and feverish, pulse very rapid, breathing free and perfectly easy, but eighty respirations to the minute. Warm flax-seed poultices were applied to the chest and back, and covered with oiled silk. The little patient died at 9 A. M., six days after intubation.

Shortly after death a post mortem was obtained. Upon removing the trachea and larynx, the tube was found in position and unobstructed. There was slight swelling at the base of the epiglottis, mucous membrane of the larnyx clear and not greatly thickened. Some false membrane was found in the trachea, but very loosely attached to the mucous membrane. Complete hepatization was observed of the upper lobe of left lung, while the lower lobe of both lungs were greatly congested and more or less hepatized. The small ramifications of the bronchi were filled with opaque mucus.

It is with pleasure that I report intubation of the larynx a success. It is an equal pleasure to know that this advance, this great achievement, has been the result of patient labor, the skill and courage of an American physician. I predict that at no distant day tracheotomy will in a great measure be superseded by this simple, safe and bloodless operation.

3449 Indiana Ave.

